# Introduction: Development, Young People, and the Social Production of Aspirations

**DOI:** 10.1057/s41287-020-00337-1

**Published:** 2020-11-19

**Authors:** Roy Huijsmans, Nicola Ansell, Peggy Froerer

**Affiliations:** 1 International Institute of Social Studies of Erasmus University Rotterdam, Kortenaerkade 12, 2518AX The Hague, The Netherlands; 2 Department of Social and Political Sciences, Brunel University London, Kingston Lane, Uxbridge, UB8 3PH UK

**Keywords:** Aspiration, Childhood, Development, Research, Temporality, Youth, I0

## Abstract

In this editorial introduction to the Special Issue Youth, Aspirations and the Life Course: Development and the social production of aspirations in young people’s lives, we put the work presented in this collection in conversation with the wider literature on development, youth and aspirations. Aspiration we define as an orientation towards a desired future. We elaborate on our conceptualisation of aspirations as socially produced and reflect on the methodological challenges in researching young people’s aspirations in development. While mindful of the various critiques of aspiration research we argue that aspirations constitute fertile terrain for theorising the temporal dynamics of being young and growing up in contexts of development.

## Résumé

Dans cette introduction éditoriale au numéro spécial “Jeunes, aspirations et parcours de vie: développement et production sociale des aspirations dans la vie des jeunes”, nous réalisons un parallèle entre le travail présenté dans cette collection et la littérature consacrée au développement, à la jeunesse et à ses aspirations. Nous définissons “aspiration” comme une orientation vers un avenir souhaité. Nous abordons plus amplement la façon dont nous conceptualisons les aspirations comme étant produites socialement et nous réfléchissons aux défis méthodologiques auxquels fait face la recherche sur les aspirations des jeunes dans le cadre du développement international. Tout en étant conscients des diverses critiques visant la recherche sur les aspirations, nous soutenons que les aspirations constituent un terrain fertile pour théoriser la dynamique temporelle de ce que signifie être jeune et grandir dans un contexte de développement international.

## Defining Aspirations

Development discourse is thick with aspirations (Frye 2012; Jakimow 2016). Planning for and bringing about desired forms of change is key to development practice and the longing for a better life constitutes a main driving force for people’s continued engagement with development interventions despite its all-too-frequent disappointments (De Vries 2007; High 2014; Li 2007). Reflecting trends in the Global North (e.g. Baillergeau and Duyvendak 2019; St. Clair et al. 2013; Stahl et al. 2017), the concept of aspiration features most explicitly in the development studies literature focusing on the relation between education and young people’s ideas about the future (e.g. Frye 2012; Morrow 2013; Naafs and Skelton 2018). Yet even in these studies, aspiration too often remains an elusive concept. This is not least because in many languages the word for ‘aspiration’ is not employed in everyday speech, rendering the connection between empirical reality and academic abstraction highly ambivalent. For example, in our research in rural, remote Laos we never heard the Lao language word for aspirations (*khwaampaathanaa*) being used spontaneously.^1^ Even the teachers and district level staff of the Ministry of Education, when asked, had to think about the correct Lao language word for aspirations. More often, aspirations were referred to in terms of ‘hope’, ‘wish’, ‘want’, or ‘dream’. To complicate matters further: at times, these different words were indeed used to emphasise differences in how one relates to a desired future. In other instances such terms were used interchangeably.

The academic literature on aspirations is equally ambivalent in its use of terminology. For example, while some studies use *aspirations* and *expectations* interchangeably other authors insist on making a conceptual distinction (Leavy and Smith 2010). Frye (2012, p. 1566) explains she uses ‘expectations’ to refer to ‘concrete plans and probable scenarios’ signalled by her respondents’ use of words such as ‘”plan,” “think,” and “expect”’, and the term ‘aspirations’ ‘for statements related to ideals or desires’ indicated by words such as ‘”want,” “desire,” “hope,” and “aspire”’. While Frye distinguishes expectations from aspirations, she conflates the latter with desire, something that, again, other scholars take issue with (e.g. Carling and Collins 2018; High 2014). For example, drawing on Deleuze and Guattari, Collins (2018, p. 966) explains that the concept of desire directs our attention also to embodied and emotional dimensions, whereas the concept aspiration remains more often limited to the conscious and cognitive realm (see also Rodan and Huijsmans, this issue).

In this Special Issue, we use ‘aspiration’ to refer to *an orientation towards a desired future*. Such futures may be individual or collective projects, may refer to more immediate or longer term futures and include both the work of imaginations and affect as well as material practices. We recognise that these orientations may well be called ‘drafts’ (Du Bois-Reymond 1998, p. 64) because they are subject to constant revision. Further, while the future is unavoidable and a serious matter (Zipin et al. 2020, p. 2), for most young people an aspired future ‘clearly will never become reality in the way it was planned’ (Du Bois-Reymond 1998, p. 64).

Another key element to our working definition is the term ‘orientation’. An orientation towards the future is an active engagement with it, including imagining possibilities, doubting trajectories, and navigating the relations through which futures unfold. Through this work, the future gains an affective, or more consciously, a cognitive presence in the present, and the quest of realising a certain future becomes situated in a social landscape. Orientations may direct young people towards concrete actions in order to realise desired futures, towards acts that amount to keeping such futures open (see Huijsmans et al. this issue) and, perhaps most importantly, orientations may take the form of struggles with and disappointments about obstacles ‘to what, in past contexts, might have been reasonable strategies for pursuing futures; and …to imagining alternatively viable trajectories into futures’ (Zipin et al. 2020, p. 2).

Finally, following Frye (2012) and Johnson-Hanks (2005) we agree that especially in contexts of rapid change characterised by high levels of uncertainty, we need to be extremely careful in connecting orientations towards the future with the logic of rational choice models that link actions in the present to concrete outcomes in the future. Therefore, rather than seeking to distil the future from young people’s aspirations several of the contributing articles (e.g. Dost and Froerer; Kaland; Rodan and Huijsmans; Rumsby; Van Stapele) illustrate that orientations towards the future are more productively understood as ‘assertions of identity’—refinements of young people’s narratives about themselves whereby they seek to ‘transcend their present reality’ (Frye 2012, p. 1567).

## Conceptualising Aspirations as Socially Produced

By viewing aspirations as socially produced, this collection builds on Appadurai’s premise that ‘aspirations are never simply individual’ and ‘always formed in interaction and in the thick of social life’ (Appadurai 2004, p. 67). This directs attention to institutions and social relations, as well as to everyday practices and specific encounters giving rise to aspirations, transforming them or contributing to their waning. We bring this premise into dialogue with a relational perspective towards understanding children and young people in development (Huijsmans 2016; Huijsmans et al. 2014). This approach emphasises that age-based categories and generations are relational constructs, it recognises young people’s situated agency in these sets of relations, and appreciates development as an intervention in the relations through which childhoods and youths are (re)constituted and realised as lived experiences.

The various articles comprising this Special Issue combine these two perspective in their analysis of research findings generated with very differently situated groups of children and young people—ranging from young men in a Nairobi ghetto (Van Stapele, this issue) to highly educated young Portuguese migrant women in London (Rodan and Huijsmans, this issue). On that basis, the articles zoom in on the specific social interactions and encounters through which people come to ‘sense’ the viability of specific sets of aspirations, including how these may incite ‘a will to believe and act’, or ‘wane’, as young people live them over in changing social-life contexts brought about by the intersection of life course dynamics and the broader dynamics of social change (Zipin et al. 2020, p. 2). For the de facto stateless ethnic Vietnamese children in Cambodia, Charlie Rumsby’s contribution focuses on, this included being recognised as ‘good children’, for example, when picking up rubbish in their neighbourhood as well as the alternative futures they came to sense through the collective practice of prayer.

Our focus on the social production of aspiration is premised on sociological and anthropological theory which we will briefly introduce below. Thereby, the Special Issue can be read as a critique to development studies literature using the concept of aspirations without much theoretical grounding [as is common in policy publications (e.g. FAO, IFAD and CTA 2014)] or that is premised on a conceptualisation of aspiration rooted in behavioural economics, neuroscience and social psychology. The 2015 World Development Report entitled *Mind, Society, and Behavior* (World Bank 2015) is an influential example of the latter. Although it pays lip service to sociological and anthropological conceptualisation of aspiration and acknowledges that such an understanding would complicate any quick policy solution because it requires ‘influencing not only the cognitive decision making of particular individuals but also social practices and institutions’ (World Bank 2015), its main gist is that tweaking ‘mental models’^2^ is a promising and relatively inexpensive addition to the development practitioner’s toolkit. This approach to tackling development problems takes the form of ‘nuances in design or implementation […] labelling something differently […] offering reminders, activating a latent social norm, or reducing the salience of a stigmatized identity’ (World Bank 2015, p. 3) and is illustrated with numerous examples from empirical studies. A typical example is a study conducted in Ethiopia in a context in which ‘disadvantaged individuals’ are said to commonly describe themselves as neither having a dream nor imagination, and to claim that they only live for today (World Bank 2015, p. 4). This mental model was altered by having people watch ‘an hour of inspirational videos comprising four documentaries of individuals from the region telling their personal stories about how they had improved their socio-economic position by setting goals and working hard’ (ibid 2015: 4). Six months after the intervention those who had watched the documentaries ‘had higher total savings and had invested more in their children’s education, on average’ (ibid 2015: 4). In short, the videos had raised people’s aspirations leading poor people themselves to realise pre-defined development outcomes.

Our critique of development interventions that revolve around the idea of ‘raising aspirations’ is based on the premise that such interventions effectively promote ‘individual investment in institutional pathways for self-improvement while protecting these same institutions from blame when these pathways do not lead to the imagined outcomes’ (Frye 2019, p. 724).^3^ Several of the contributing articles discuss these and other problems associated with aspiration research and aspiration based development policies. Ben White’s contribution is perhaps the most articulate in this regard. He makes clear that when aspiration research is reduced to a focus on the mind and is translated into interventions that merely seek to tweak ‘mental models’, we are back at a long-standing, yet problematic strand of development research which White aptly summarises as ‘blaming poverty on defective aspirations’.

Sociological and anthropological conceptualisations of aspirations situate the production of aspiration as part of the social. This means that aspirations may manifest at the level of the individual, but cannot be reduced to it. The work by Zipin et al. (2015) theorises this in more detail. Drawing on the Bourdieusian concepts of doxa and habitus, Zipin et al. (2015) distinguish between doxic and habituated social logics. The former refers to aspirations rooted in discourse and practices that have acquired ‘a taken-for-granted status’ but are in fact ‘grounded in *populist-ideological mediations’* (p. 231, original italics). Zipin et al. (2015, p. 232) illustrate this with reference to the ethic of ‘If you work hard enough you can attain your dream’.^4^ Young people are exposed to such doxic logics in the context of school but also through media, religion, popular culture and the market (i.e. populist-ideological mediations). Moreover, international development agendas are rife with doxic logics such as the idea that GDP growth leads to development, and in relation to children and youth such doxic logics are evident from the continued, and largely unquestioned, emphasis on human capital in realising school-to-work transitions (White, this issue; World Bank 2006). The two contributions on China in this Issue (Kaland; Sier) underscore that doxic logics producing aspirations are also central to national development agendas, and that these carry a power of symbolic violence because ‘they codify the norms, and so select for the success, of those in relatively powerful positions, yet hold sway among others—whose lack of success thus appears justified as a the result of ‘deficits’, or ‘lacks’, of aspiration’ (Zipin et al. 2015, p. 231).

Aspirations produced through habituated logics, Zipin et al. (2015, p. 234, original italics) describe as ‘possibilities-*within-limits* of given social-structural positions’. Several of the contributions show that as young people grow up and become aware of their gendered, ethnic, and/or class- and caste-based position in society, certain ideas about the future become seen as out of reach while others are deemed realisable ‘for the likes of us’. We see a habituated logic at play when Rudi, a sharecropper from Yogyakarta, Indonesia quoted in the contribution by Huijsmans et al. realised that coming from a poor background it would be impossible for him to ever become a police officer or a (local) government official. This led him to say he wanted to become a farmer when he was asked by his teacher despite not much liking the farm work he had to do as a child. However, the contribution by Rodan and Huijsmans shows that in contexts of international migration, young people may also mute these biographic dimensions in the stories of self they construct and enact and thereby seek to build non-habituated aspirational subjectivities.

Next to these Bourdieusian approaches to understanding aspirations, Zipin et al. (2015, p. 238) also propose the idea of emergent aspirations, which they describe as ‘imaginings, voicings and agentic impulses towards alternative futures—neither doxic nor habituated but reimaginative in their logic’. We suggest that emergent aspirations have particular purchase in contexts of development, which are not only locales thick with aspirations (discussed in more detail below) but also characterised by rapid change (Naafs and Skelton 2018, p. 5). For example, due to urbanisation and migration a good part of the current generation of youth in the Global South lives in different localities than their parents’ generation, is on the whole is more educated (see Kaland, this issue; Sier, this issue; Naafs and Skelton 2018), and the technological and media landscapes young people grow up in and co-construct are also radically different compared with just a decade ago, even in remote rural locations (Amankwaa et al. 2020; Huijsmans and Trần 2015). For these reasons, the past (or the parental generation) cannot be assumed to work as a reliable guide for the future as the habituated logic towards understanding aspirations implies. And in contexts characterised by weak institutions (see Rumsby, this issue) or for social groups that live on the very margins of society and its mainstream institutions (see Van Stapele, this issue), neither can we assume that doxic aspirations necessarily hold much sway apart from giving rise to representations casting these young people as non-aspirational, or even worse as waste or failures.

A key contribution of this Special Issue is that growing up in marginalised social environments characterised by poor quality education, lack of citizenship, precarious forms of migration or even structural violence causing an extremely low life expectancy does not mean that young people are deprived of the kind of social interactions that give rise to emergent aspirations. Even in such marginalised conditions young people still ‘apprehend the present-becoming-future’ (Zipin et al. 2015, p. 236). The papers in this issue show that in such adverse conditions orientations towards the future are subject to frequent setbacks and that simply maintaining an orientation or refocusing it is no small feat and essential for making life meaningful. Or in the words of one of the young men featured in Naomi van Stapele’s contribution: ‘Without hope you are already dead’.

## Development and Aspirations in Young People’s Lives: Back to the Future?

On first sight, a concern with aspirations appears in tension with one of the central premises of the social studies of childhood and youth. In international development, a focus on children and youth was for long, and too often still is, motivated by the returns that investments in children and youth would generate when these young people would become adults—e.g. in terms of labour productivity, the kind of citizenship they would enact, the kind of motherhood and fatherhood they would practice. The social studies of childhood and youth constituted a critique to an interest in children and youth based on what young people would eventually become. It pleaded for a shift in focus: away from young people as adults-in-becoming towards a focus on young people in their present condition as children and youth. In development studies, the influence of the social studies of childhood and youth can be recognised in the emergence of so-called child- and youth-centred perspectives on issues such a poverty (e.g. Camfield 2010), work (Bourdillon et al. 2010), and migration (Huijsmans and Baker 2012).

Since aspirations are necessarily future oriented, the approach set out in this Special Issue directs attention to the temporal dimension of being young. It illuminates the role of the future in young people’s present lives, and seeks to tease apart the extent to which young people’s orientations towards the future are grounded in their biographical past, shaped by key events in the present, and influenced by ideas about the future flowing from development agendas (e.g. Dungey and Ansell). For example, the practice of schooling as a key component of (inter)national development agendas places a firm temporal rhythm on the experience of being young. This is brought about by the age-graded structure of most school systems, the supposed progression through several stages of the school system towards an assumed occupational endpoint, but also by minute details such as the structuring of the school day on the basis of clock time. In addition, through the curricular design influential international development actors as well as national governments steer young people towards particular orientations of the future (Ansell et al. forthcoming; World Bank 2015, pp. 70–71). The silences and emphases in the educational design and delivery give certain futures a normative status and render these desirable while alienating children and youth from possible others (Dungey and Ansell 2020; Huijsmans and Piti forthcoming). Everyday participation in schooling gives the future a firm presence in children and young people’s everyday lives. A focus on aspiration provides ground for theorising the various temporalities shaping young lives and thereby expands the temporal scope of childhood and youth studies (Ansell et al. 2013).

Taking temporality seriously in the social production of aspirations during young lives also means paying attention to life course dynamics and key life course events. For example, the contribution by Huijsmans et al. shows how key events during the life course, including schooling, leaving school, marriage and migration affect the kinds of orientations towards desired futures rural youth in India and Indonesia are able to maintain, renegotiate, or need to reimagine because certain pathways are closing down on them. White makes a similar point by arguing that we should be careful in reading youth’s out-migration away from rural areas and farm-based work as evidence of their lack of interest in becoming a farmer. A life course perspective, White argues, shows that these young people some years later may well return to rural areas and enter farming. Various other contributions demonstrate that key transition moments in young people’s lives, such as transitions in their school trajectory (e.g. Kaland, this issue; Sier, this issue); in their religious becoming (e.g. Rumsby, this issue), becoming a young migrant (Rodan and Huijsmans, this issue) or in relation to the social life of urban gangs (Van Stapele, this issue) constitute particularly fruitful moments for researching the social production of aspirations. It is in such instances that the present gains a particularly strong bearing on the future, which thus constitute fertile moments for researching aspirations.

## Methodological Challenges and Solutions

Tanya Jakimow (2016, p. 11) defines development contexts as ‘locales thick with the discourses, practices, and institutions of international development’. Describing the development context of Indonesia, Li (2007, p. 1) notes that ‘talk of improvement is everywhere’, as are programmes that seek to improve the condition of a specific or general population (Li 2007, p. 1). All this renders contexts of development deeply aspirational landscapes which is evident in the omni-presence and traction of terms and phrases such as improvement (Li 2007), progress and advancing (Rigg et al. 1999), moving forward (Dost and Froerer, this issue), modernity and being ‘up-to-date’ (Mills 1997). Following this understanding of development we expand the scope of development studies beyond the conventional ‘developing world’. As the contribution by Rodan and Huijsmans illustrates, Portugal’s recent history fits well such an understanding of development with the European Union and the International Monetary Fund acting as a key agents of Portugal’s recent development trajectory affecting social life in Portugal in both material and discursive ways.

Understanding development contexts as social landscapes imbued with aspirations, poses a major challenge for researching aspirations. How does one avoid producing research that uncritically reproduces normative discourse on aspirations? This challenge is especially acute for research with children and youth who are often accessed by researchers through the school (see several of the contributions in this Special Issue). Since schools are a key mechanism through which normative aspirations are channelled, there is a danger that research conducted in schools ends up reproducing the dominant norm, a problem that is further aggravated by the fact that researchers themselves often embody this norm (i.e. highly educated people in salaried employment, who are often urban based and might also differ in racialized ways^5^).

Ben White’s contribution to this Issue shows that much research on young people’s aspiration takes the form of survey research or focus group discussions. In such one-off research encounters it is virtually impossible to assess the extent to which research results are merely appropriate answers based on dominant norms—and, research conducted in group settings among peers is known to lead to precisely such answers (Smithson 2000). In his discussion of various research exercises on rural youth’s aspirations White flags a marked difference between findings generated through an anonymous SMS survey and results obtained through face-to-face research interactions. This point underscores the importance of reflecting critically on issues of positionality and context in the production of knowledge about young people’s aspiration—something that all contributors have made a point doing in their respective contributions.

Mindful of the methodological pitfalls in learning about aspirations on the basis of one-off research encounters (e.g. survey research, focus group discussions) the articles in this Special Issue all draw on qualitative research (qualitative interviewing, ethnography, participatory research, and visual research) comprising multiple research encounters (the exception is White’s contribution which is a critical analysis of secondary data). This allowed developing a level of rapport necessary for going beyond normative discourses. In addition, multiple research encounters with key research subjects (including with relevant others such as parents, friends, teachers), varying the use of methods, conducting research in multiple settings and in telling moments, and sustaining research relations over time not only generates more, and more complex data, it also helps building frames of interpretation that are not just derived from the literature but also make sense in relation to the particularities of the specific research context. Thereby the articles go beyond reporting and describing aspirations towards theorising the way aspirations are produced through sets of social relations that are always situated in particular ways and grounded in particular contexts and (life)histories. Capturing the full richness of these dynamics meant privileging qualitative depth over quantitative breadth in the presentation of the data. In three of the contributions this even took the classical anthropological form of providing thick description of just one or two cases (Rodan and Huijsmans, this issue; Sier, this issue; Van Stapele, this issue).

Finally, capturing the temporal dimension of the social production of aspiration during young people’s life courses was achieved in various manners. Huijsmans et al. did so by using life history interviews as a key method in their research with rural youth and young adults (see also Afutu-Kotey et al. 2017, pp. 481–482). Working with children, Rumsby opted for visual research methods in order to understand ‘how participants see themselves in the present, what significant events have shaped their past and how they imagine their future’ (Rumsby, this issue). Again, other contributions captured the temporal dimension of the social production of aspiration by drawing on long term research relations, in the case of Van Stapele spanning many years.

## The Contributing Articles

The Special Issue is composed of eight original articles and this editorial introduction. Five articles (Dost & Froerer; Huijsmans et al.; Kaland; Rodan & Huijsmans; White) have come out of a conference organised at Brunel University London, United Kingdom in March 2018 entitled *Theorising young people’s aspirations in a global context: An interdisciplinary conference*. Three articles (Rumsby; Sier; Van Stapele) joined in later.

Each contributing article can be read as a stand-alone piece but here we emphasise a number of threads running through the issue as a whole. First, all contributing articles engage critically with the premise often found in development literature of placing much hope on young people for realising development aspirations (Camfield 2011, p. 669).^6^ Conceptualising aspirations as socially produced means taking seriously children and youth as aspiring subjects who are capable of envisioning, enacting, and bringing about social change (e.g. Castañeda 2012; Cole and Durham 2007, p. 18; Jeffrey and Dyson 2016), but also the Foucauldian logic of youth as a site of intervention for realising pre-defined development aspirations (e.g. Ansell et al. 2012; Bessant 2003; Sinha-Kerkhoff 2011). The articles question the common idea of children and youth as inherently aspiring subjects simply because they are young and have most of their lives still ahead of them (and Van Stapele’s contribution underscores that the latter should not be taken for granted either). Equally, while recognising the influence of institutions such as the school and the family in shaping young people’s aspirations (Cole and Durham 2007, p. 8) the contributions show that this is not to say that social forces determine aspirations.

Secondly, all articles recognise the intersectional condition of childhood and youth. That is, young people are never just children or youth. Their lives are constituted through intersecting sets of social relations, which in addition to age include gender, class, ethnicity, religion, and caste to mention just some. For example, Van Stapele’s contribution is based on research with young men and their aspirations cannot be understood without attending to local ideas about masculinity, especially with respect to becoming a provider for their family as they move from the social position of junior men to senior manhood. In Rumsby’s article it is religion and nationality that, in a triad with age, are the key social relations in the production of aspiration. Class features prominently in the two articles based on research with youth in China (Kaland, this issue; Sier, this issue) while it constituted an important absent present in narratives of migration constructed by the young Portuguese migrants in London featured in the contribution by Rodan and Huijsmans.

Thirdly, migration plays a prominent role in a number of articles. In the contribution by Willy Sier (this issue) this takes the form of focusing on the social production of aspirations among China’s education migrants; youth who have migrated from rural to urban China via the country’s higher education system. In contrast, Ole Kaland’s article is based on research with children of Chinese internal migrants who attend Shanghai’s vocational education. These youth have grown up in urban China, yet their household registration ties them to provincial China which has severe consequences for their educational opportunities and complicates the very idea of where and how to build a future. Kaland argues that in contrast with the widespread sentiment that casts these youth as non-aspirational, the adverse structural condition they face leads these youth to ‘become more aware of their options and develop more pronounced orientations towards their future than their local and middle-class peers do’. Moreover, their aspirations are less driven by the normative idea of the good life, and more by a pragmatic desire for ‘a better life’. Whereas Kaland writes about migrant youth who have decided not to try and get into university education, Sier focuses on education migrants who *did* enter university in Wuhan, Hubei province, China (see also: Sier 2020). She emphasises the high price that these educational migrants pay for realising China’s national aspiration of educational expansion. Her contribution also describes the double edged sword educational aspirations hold in the context of China’s transition. For young people and their families university education holds the promise of upward social mobility. Yet, materially it drives rural youth to the cities, ‘loosening the next generation’s grip on agricultural land, and facilitates another wave of urban–rural wealth extraction through educational fees’.

Migration also features prominently in the lives of the ethnic Vietnamese children who are the focus of Rumsby’s contribution. They are born in Cambodia but without official birth registration and Cambodian nationality they are de facto stateless. As Rumsby shows, this delimits their opportunities in Cambodian society as they are not able to complete their education in Cambodian state schools. Yet, it is also generative of emergent aspirations triggered by the various religious and non-religious activities they participate in through the ‘God School’. Collectively, the contributions by Kaland, Rumsby and Sier shed light on the interplay between education and migration in the production of aspirations.

In the contributions by Lisa Rodan and Roy Huijsmans (this issue) and Huijsmans et al. (this issue) it is young people themselves who become migrants. For one of the young highly educated Portuguese migrants in London discussed by Rodan and Huijsmans (this issue), migration is key to her performance of a highly aspirational form of personhood linked to discourses about the cosmopolitan Eurostar generation discourse which she used to distinguish herself from earlier generations of Portuguese migrants. As she put it: ‘those people [earlier generations of Portuguese migrants] they had less education, they migrated as they had no opportunity to work. It wasn’t about personal development’ (Rodan and Huijsmans, this issue). For the rural youth discussed by Huijsmans et al., migration is aspirational too but rather as a means to keep options open and to delay entry into farming on a full-time basis.

A fourth thread running through the collection is that all articles focus on what aspirations mean and how they are produced among groups of children and youth that are marginalised to various extents. Marginalisation is a relative concept. It holds true for the middle-class youth coming of age in post-austerity Portugal that Rodan and Huijsmans write about for whom there is little prospect of finding professional recognition in the Portuguese labour market which is most strongly expressed through the *geração a rasca* (desperate generation) discourse that one of the protagonists associates with. However, this is evidently a very different degree and form of marginalisation compared to the young men growing up in the Kenyan ghetto neighbourhood Van Stapele writes about. For them, reaching the age of twenty-five is already a major aspiration. In a context of structural violence, this cannot be taken for granted. It requires ‘having a focus’ and ‘deciding to have a future’ (Van Stapele, this issue). Marginalisation also aptly describes the social condition of the *adivasi* children and youth in rural Chhattisgarh, India, Arshima Dost and Peggy Froerer (this issue) write about. As Dost and Froerer argue, for rural *adivasis* education is key to the aspiration of moving forward while it also channels young people into a very narrow set of pathways through which to realise progress.

In the contributions of White (this issue) and Huijsmans et al. (this issue) marginalisation features in a different way. In these two articles, the focus is on an aspiration that has become marginalised by the aspiration regimes characterising present-day development. It concerns the aspiration of becoming a small-holder farmer. The two articles approach the issue differently. White’s contribution is a sceptical reading of policy research about the aspirations of rural youth in relation to farming futures. He provocatively asks what influential agents of rural development such as FAO and IFAD^7^ ‘know about young rural men and women’s aspirations’ and how this compares with findings from more in-depth, ethnographic studies with rural youth. Based on research in India and Indonesia, Huijsmans et al.’s contribution shows that farming continues to play a role in the lives of rural children and youth but that few aspire to becoming a full time farmer at a young age. They argue that rural youth’s aspirations are often broad and fluid and that such orientations towards the future lead to a strategy of keeping things open (through schooling, through migration, by farming part-time only, and for some young men through marriage). The reluctance to get into full time farming too early, Huijsmans et al. argue, is because being fully occupied with farming in rural areas is perceived as closing off too soon any possibility of an alternative future.

As a whole, the Special Issue seeks to make a balanced intervention in research and practice concerning aspirations in relation to young people and development. We have highlighted the critique of interventions seeking to realise development objectives by ‘raising aspirations’. Yet, rather than dismissing a focus on aspirations as distracting attention from relations of power, deep-seated inequalities and the working of institutions, we have made the case for taking aspirations seriously in development studies not least because the social landscapes of development we have described are deeply aspirational. This required grounding the ambivalent concept of aspirations theoretically as part and parcel of the social rather than a mere mental model that can easily be tweaked. We have done so by conceptualising aspirations as socially produced, recognising how its production is always situated in particular histories and places, politico-economic presents and mediated by cross-cutting relations including, but not limited to, class, gender, and generation. In relation to young people, this conceptualisation highlights that the work of aspirations is an ever unfolding dynamic through which possible futures and particular pasts come to have a bearing on the lived moment of the present. Gaining some control over this, however fragile or illusive this may be, is key to making life meaningful, perhaps especially so in adverse and rapidly changing circumstances.

## Footnotes

1 The Special Issue is conceived as part of the project ‘Education systems, aspiration and learning in remote rural settings’. It received funding through the ESRC-DFID scheme Raising Learning Outcomes in Education Systems (ES/N01037X/1). For this project, field research was conducted in remote, rural India, Laos and Lesotho. For further details: https://www.brunel.ac.uk/research/Projects/Education-aspiration.

2 Defined as ‘one’s belief in what is possible in the future’ (World Bank 2015, p. 4).

3 In line with this critique we also avoid the normative language of ‘low’ and ‘high’ aspirations.

4 Note how this echoes the idea of ‘those who work hard will succeed’ that Sier (this issue) encountered in her research in China.

5 Note for example Frye’s (2012, p. 1581) methodological strategy ‘to minimize the effects of the interview setting’, in her research on aspirations among young female secondary school students in Malawi by working with ‘young women [research assistants] who were as close as possible to the status of the respondents, residing in nearby towns and possessing no postsecondary credentials’.

6 See also the 2007 World Development Report that focused specifically on youth (World Bank 2006).

7 FAO is the Food and Agricultural Organisation of the United Nations, IFAD is the International Fund for Agricultural Development which is also a United Nations’ agency.
